# Newborn Screening for Sickle Cell Disease and Other Hemoglobinopathies: A Short Review on Classical Laboratory Methods—Isoelectric Focusing, HPLC, and Capillary Electrophoresis

**DOI:** 10.3390/ijns4040039

**Published:** 2018-12-05

**Authors:** Claudia Frömmel

**Affiliations:** 1 MVZ Alexianer Labor GmbH, Groβe Hamburger Str. 5–11, 10115 Berlin, Germany; c.froemmel@alexianer.de; Tel.: +49-30-2311-2820; 2 Newborn Screening Laboratory, Charité-Universitätsmedizin Berlin, Augustenburger Platz 1, 13353 Berlin, Germany

**Keywords:** newborn screening, sickle cell disease, hemoglobinopathy, laboratory methods, neonatal screening, hemoglobin pattern, HPLC, IEF, capillary electrophoresis

## Abstract

Sickle cell disease (SCD) and other hemoglobinopathies are a major health concern with a high burden of disease worldwide. Since the implementation of newborn screening (NBS) for SCD and other hemoglobinopathies in several regions of the world, technical progress of laboratory methods was achieved. This short review aims to summarize the current practice of classical laboratory methods for the detection of SCD and other hemoglobinopathies. This includes the newborn screening technologies of high-performance liquid chromatography (HPLC), capillary electrophoresis (CE), and isoelectric focusing (IEF).

## 1. Introduction

Newborn screening (NBS) for sickle cell disease (SCD) as one element of prevention programs for hemoglobinopathies has been in place in several regions in the world for more than 40 years now. First attempts were made in the United States of America (USA) and Jamaica, as well as in Great Britain in the 1970s [1,2]. Starting with alkaline electrophoresis, the development of faster and more precise methods permitted the development of extended and more sophisticated prevention programs of hemoglobinopathies. The aim of NBS for SCD is to prevent major health complications of SCD from early childhood onward. SCD in this context comprises all forms of relevant sickling disorders, originating from different genetic constellations (e.g., homozygous HbS (HbSS) and compound heterozygous forms as HbS/β^0^ thalassemia, HbS/β^+^ thalassemia, HbS/HbC, HbS/HbD^Punjab^, HbS/HbE, HbS/HbO^Arab^, HbS/Hb Lepore, and HbS/δβ thalassemia). As life-threatening events can occur in children with SCD from three months of age onward, early diagnosis is desirable to establish preventive measures. Patients with thalassemia and other major hemoglobin disorders do not benefit in the same way from early diagnosis. However, these disorders have important implications for family health, and the burden of disease is very high in several regions of the world, thus demanding preventive action. In this context, many newborn screening programs make use of the possibility to detect carriers of hemoglobin variants as a by-product to provide families with reproductive knowledge and informed decision-making regarding hemoglobinopathies.

Laboratory methods to detect disease states and carrier states (if included in the program) should be very sensitive and highly specific. To prevent harm, they should not miss patients and should not falsely identify patients. Psychosocial and logistic burdens to families from screening or diagnostic evaluation, an increased risk of unnecessary medical treatment, or a delayed diagnosis from false negative results, as well as psychosocial harm from false positive results and uncertainty of clinical diagnosis, should be minimized and be outweighed by the benefits [3].

While laboratories for newborn screening for endocrine and metabolic disorders mainly use tandem mass spectrometry (MS/MS) or photometric assays, technologies, which are commonly established for NBS for hemoglobinopathies include HPLC, isoelectric focusing (IEF), and capillary electrophoresis (CE). They were developed for larger sample sizes from routine hemoglobinopathy testing and permit the clear separation of hemoglobin variants of interest in an automated or semi-automated way. Recent developments of screening methods like tandem mass spectrometry (MS/MS) and Matrix-assisted laser desorption/ionization time-of-flight mass spectrometry (MALDI-TOF MS) or molecular genetic testing are explained elsewhere.

## 2. Technologies

Technologies are based on the separation of hemoglobin species (Hb) and the quantification of respective hemoglobin fractions from dried blood spot (DBS) or fresh cord blood samples. They should be able to detect HbS and HbC, and separate HbF and HbA, as well as being able to detect other hemoglobin variants of clinical relevance, such as HbD^Punjab^, HbE, Hb Lepore, and HbO^Arab^. The screening methods reviewed here provide provisional results and identify putative abnormal fractions of hemoglobin and have to be confirmed to give a definite diagnosis.

In contrast to the diagnosis of carriers and patients with hemoglobinopathies in clinical routine or carrier screening programs, where a complete blood count with erythrocyte indices and parameters of iron metabolism are included, newborn screening is only based on the separation of hemoglobin from DBS or cord blood. Additional tests such as the solubility test for HbS are not applicable. As symptoms usually do not occur before two months of age, results of screening should be provided within this period. An algorithm designed to clearly state when parents should be informed and a defined follow-up help avoid reduced effectiveness of the whole process.

### 2.1. HPLC

**Figure 1:**
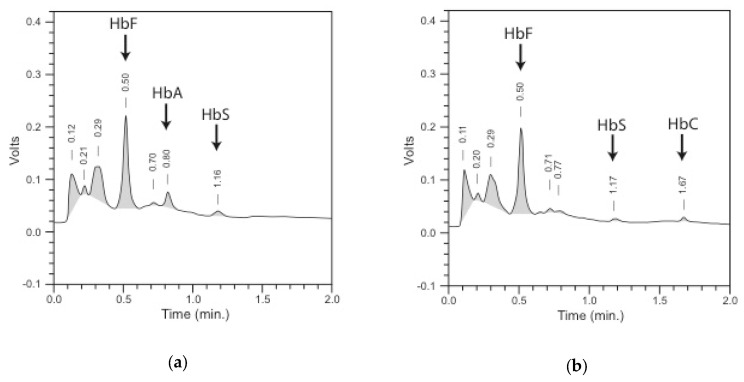
Chromatogram of HPLC (Variant™ newborn screening (nbs), Bio-Rad laboratories, Europe): the *x*-axis represents the time in minutes, and the *y*-axis represents the response in volts; retention times of integrated peaks are shown above the peaks, and peaks of Hb variants included in the pattern are named and indicated with an arrow: (**a**) pattern of HbF/HbA/HbS (FAS); (**b**) pattern of HbF/HbS/HbC (FSC).

Automated cation-exchange high-performance liquid chromatography (HPLC) is a widely used method to screen for hemoglobinopathies with a clear separation and quantification of hemoglobin fractions [4,5,6,7,8,9,10,11]. The method is based on the elution of hemoglobin bound to a solid phase over time by buffers with a pH gradient. The time from the injection of the sample into the column to the elution of the hemoglobin fraction is called the retention time. Eluted hemoglobin is detected by a dual-wavelength detector and quantified by integrating the area under curve of the produced chromatogram, expressed as a percentage of the total area (Figure 1). A characteristic retention time (window of detection), together with the relative quantification, permits presumptive identification of all relevant hemoglobin species: HbF, HbA, HbA_2_, HbS, HbC, HbD^Punjab^, HbE, Hb Lepore, and HbO^Arab^. Hb Bart’s is also detected. HPLC analysis is very precise and reproducible with an inter-run coefficient of variation (CV) of <5%. The detection limit for HbA and HbS was shown to be around 1% of total hemoglobin [4,6,12]. Calibrators and material for internal quality assurance containing Hb FAES and FADC (where letters represent different Hb species) are available.

### 2.2. CE

**Figure 2:**
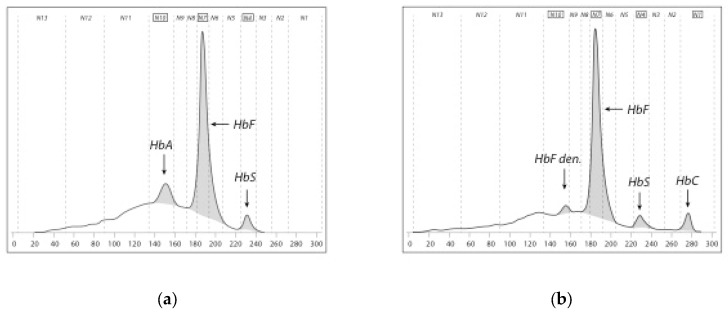
Pherogram of capillary electrophoresis (CE; Capillarys™ neonat fast, Sebia, France); zone from left to right: N13–N1. Peaks of Hb variants included in the pattern are named: (**a**) pattern of FAS; (**b**) pattern of FSC.

Capillary electrophoresis (CE) combines two principles of separation of hemoglobins, the electrophoretic mobility in alkaline buffer and the electro-osmotic flow resulting in excellent separation. High voltage applied to an in silica glass capillary prompt hemoglobin molecules to migrate toward a detector of 415-nm wavelength. Detected hemoglobin fractions can be relatively quantified and produce a pherogram. HbF is centered in the neonatal system (note: it is HbA in the adult system), followed by the automated integration of peaks and defined migration zones (N1 to N13—specific to the neonatal system) which allow the easy interpretation of pherograms and help identify hemoglobin patterns (Figure 2).

CE is able to detect and relatively quantify HbF, HbA, HbA_2_, HbS, HbC, HbD^Punjab^, HbO^Arab^, HbE, and Hb Lepore. Hb Bart’s is also detected. Many other Hb variants may be detected including γ- and α-chain abnormalities with an overlap in the zones of the more common hemoglobins, e.g., HbD^Punjab^ and Hb Korle Bu in zone N5 [13]. Interpretation has to be diligent with regards to reporting named hemoglobin variants following the presumptive identification of the system. Controls for internal quality assurance are provided containing HbF, HbA, HbS and HbC. The detection limit for HbA and HbS was found to be around 1% of total hemoglobin.

### 2.3. IEF

**Figure 3:**
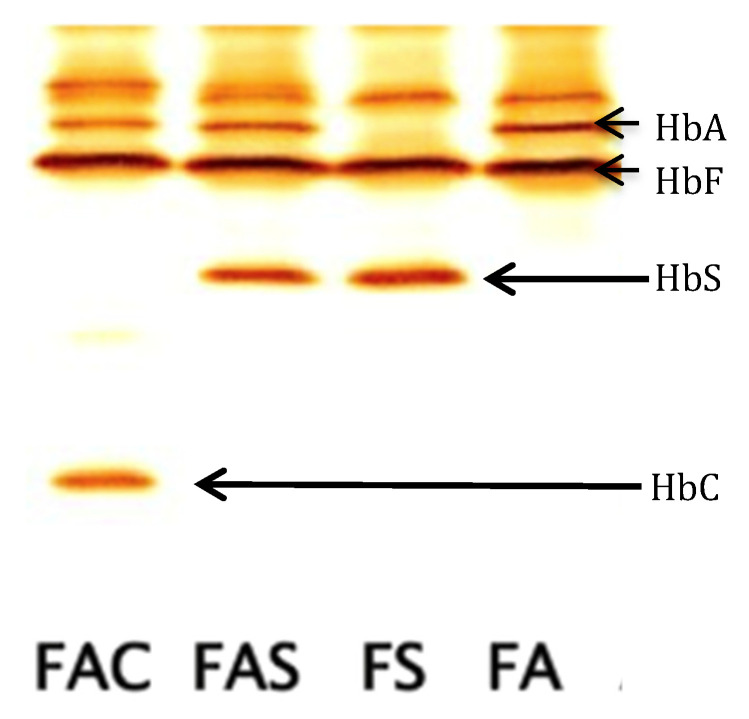
Isoelectric focusing gel picture (RESOLVE™, Perkin Elmer, Finland); from left to right: patterns of FAC, FAS, FS, and FA, adopted from Reference [14].

IEF is a very sensitive method and is widely used at relatively low costs. IEF separates hemoglobin species according to their isoelectric point on a gel medium with very high resolution. Hb variants migrate in a pH gradient to the point where their net charge becomes zero. Bands are narrow compared to classical electrophoresis and give a precise picture (Figure 3). HbF and HbA, as well as relevant hemoglobins HbS, HbC, HbD^Punjab^, HbE, and HbO^Arab^, can be separated. Hb Bart’s can also be detected. IEF separates post-translationally modified variants, which sometimes makes interpretation difficult; thus, an experienced staff is needed to read results.

IEF is a semi-automated system, which allows the parallel run of several gels entailing some labor-intensive steps. Easier work-up and documentation of gels are achieved using a scanning software. Controls for internal quality assurance contain HbF, HbA, HbS and HbC.

## 3. Interpretation of Newborn Screening Results and Hemoglobin Patterns

Normal newborn samples mainly contain HbF (α2/γ2) and a smaller amount of HbA (α2/β2). HbA levels range from 6 to 40% with an average of 19%, showing inter-individual variations. HbA levels in newborns are dependent on gestational age and date of sampling, reflecting the stage of hemoglobin switch [6,12]. In Table 1, patterns of the detected hemoglobins are listed in the order of highest percentage to the lowest. One has to be aware that relevant Hb variants may present an abnormal pattern similar to those consistent with a target disease. Today, there are more than 1000 variants of hemoglobin known. Software solutions for HPLC and CE suggest a pattern, which has to be verified by the technician reviewing the chromatogram or pherogram. In IEF, an experienced staff reviews the gel, and densitometry helps quantify hemoglobin variants to determine the pattern. Guidelines and lab handbooks can be used to interpret hemoglobin patterns, specifying the significance of a found pattern and the possible pitfalls according to the methods applied. An abnormal result with the screening method (first line) is repeated from the sample using a secondary method (second line), or using the same method if another is not available.

### 3.1. Target Diseases

Patients with sickle cell disease and non-sickling disorders, as well as carriers of abnormal hemoglobin variants, can be detected by NBS. Choosing primary and secondary target diseases influences false negative and false positive results of the overall screening program and, therefore, are important to the evaluation of the screening process. NBS for hemoglobinopathies has sensitivity and specificity close to 100% for SCD with regards to the different genetic forms defined as target diseases (see below); however, it loses sensitivity and specificity when non-sickling disorders are included. Furthermore, pre-analytic factors like prematurity and blood transfusions prior to sampling influence the sensitivity. The positive and negative predictive values vary with prevalence of the abnormal variants and thalassemia syndromes.

### 3.2. Sickle Cell Disease

Sickle cell disease is a severe hematological disorder with acute and chronic manifestations caused by a complex pathomechanism of hemolysis and recurrent vaso-occlusive events. Life-threatening early complications can be largely avoided by initiating simple and effective prophylactic measures such as penicillin, vaccination, and parent education. Primary target diseases are all common and are generally severe sickling disorders (see Table 1).

HbS/hereditary persistence of fetal hemoglobin (HPFH) is included as a very mild disorder, as it cannot be distinguished from homozygous HbS and β^0^ thalassemia/HbS. This is accepted, as the disorder seems relatively rare. The definite diagnosis should be ruled out by confirmatory diagnostics for appropriate counseling and treatment [15].

For the distinction between the disease states of HbS/β^+^ thalassemia or HbS/β^++^ thalassemia and a simple carrier state for HbS, the relative quantification of HbA and HbS and the ratio between HbA/HbS is used (mainly a cut-off of <1). This approach bears the risk of false positive and false negative results, as there is an overlap between HbS/β^+^ thalassemia or HbS/β^++^ thalassemia and a simple carrier state for HbS, especially in premature babies. In these cases, HbA is present, but at different levels depending on gestational age. Additionally, precise quantification is a problem close to the detection limit of a variant for HPLC and CE [5,7]. To standardize quantitative interpretation of screening results, cut-offs and ratios can be expressed as multiples of median (MoM) [12]. IEF is a method with a very high resolution, but it sometimes hampers precise quantification and correct classification of bands, and there is a slight tendency to over-detect variants of no significance, e.g., modified HbA or γ-variants [5,16].

For all described methods, there are difficulties due to the fact that, with over 1000 known abnormal Hb variants, some of them will migrate, differently from HbA, but together with HbS or other common variants (HbC, HbD, and HbE). Genetic constellations like Hb Hope and HbS can lead to false positive screening results for SCD, which can then be excluded by a second-line method or by confirmatory testing [17]. Unusual analytical pictures may occur, depending on whether these rare abnormal variants with migration properties similar to HbS (or other common variants) derive from γ-, α-, or β-globin chain mutations. Heterozygous α-globin chain variants produce additional peaks or bands and comprise about one-fourth of HbF and HbA (or HbS if an additional β-mutation is present) if all four α-genes are functional, or about one-third if one α-gene is missing, or one-half if two α-genes are missing, provided the α-variant is stable. From all possible genotypes for SCD, the suitable target diseases should be defined according to the whole screening process, in both the screening laboratory and the confirmatory laboratory. With regards to this definition, cases of SCD caused by very rare genotypes, such as dominant HbS with a double mutation, are missed by classical NBS [17]; moreover, milder disorders such as HbS/β^++^ thalassemia or HbS/HPFH might be detected.

### 3.3. Non-Sickling Disorders

Other hemoglobinopathies with clinical relevance, including severe disorders such as β-thalassemia major and severe α-thalassemia (HbH disease), are often also included in NBS for SCD as secondary target diseases.

#### 3.3.1. β-Thalassemia Major and Intermedia

β-thalassemia major or intermedia results from homozygous or compound heterozygous β^0^ or β^+^ mutations. Patients suffer from severe anemia, which requires regular or irregular blood transfusion and chelation therapy to prevent iron overload. Early detection is wanted to prevent babies from becoming severely anemic and to support family health in social and medical aspects by giving the possibility of informed choice for future pregnancies and by including the affected child in a comprehensive care program for thalassemia. β-thalassemia major can be expected in samples with a hemoglobin pattern of HbF only, or HbF and HbA below a certain cut-off from 0–5% (<1.5% in the United Kingdom (UK)) [18,19].

#### 3.3.2. Severe α-Thalassemia, HbH Disease

HbH disease has a variable phenotype ranging from mild microcytic anemia to severe transfusion-dependent anemia. HbH disease is caused by deletion or inactivation of three α-globin genes with an imbalance of chain synthesis, leading to the association of β-chains in adults (β2/β2), known as HbH, and of γ-chains in newborns, known as Hb Bart’s (γ2/γ2). Clinically significant hemolytic anemia, which possibly necessitates blood transfusion, can occur during fever, infections, and pregnancy. Severe aplastic crises after infection with parvovirus B19 or growth retardation during childhood, as well as iron overload in adults with or without previous transfusion, are possible complications in a minority of patients. In regions with high prevalence and also in some low prevalence areas for α-thalassemia, HbH disease is included in the newborn screening program as a target disease. Technologies such as IEF, HPLC, and CE are able to detect Hb Bart’s [20,21,22,23,24]. Several cut-offs were evaluated for HPLC or a qualitative detection of Hb Bart’s in the fraction of fast-eluting hemoglobins (≥15% or ≥20% or ≥25% for the FAST fraction on the Bio-Rad™ nbs System, evaluated on different integration principles). IEF and CE represent a qualitative analysis of Hb Bart’s. Detection of Hb Bart’s shows considerable overlap between HbH disease and heterozygous α^0^ thalassemia. Literature about the sensitivity and the specificity for Hb Bart’s is scarce. Specificity seems lower than that for β-thalassemia and positive predictive values in a low-prevalence region will be unsatisfactory. Screening policy should decide on whether false negative results outweigh false positive results or vice versa. As Hb Bart’s is unstable, sensitivity also depends on material (DBS vs. fresh-cord blood), the day of sampling, and the storage of samples until measurement.

Including HbH disease in a neonatal screening setting is still controversial, as over-diagnosis is probable [20]. It seems more acceptable in high-prevalence areas and if the definite diagnosis by verifying deletional and/or non-deletional α-thalassemia through DNA analysis is done in the screening center [10,23]. Asian publications show a higher detection rate using DNA analysis in the first line [25].

### 3.4. Carrier Detection

With IEF, CE, and HPLC, carriers of relevant Hb variants can be incidentally detected. HPLC and CE provide presumptive identification of a variant. Variant peaks occur in their respective windows or zones and, together with the percentages of HbA and the variant peak, a presumptive result can be reported. All screening programs which include carrier detection recommend two independent methods as first- and second-line laboratory methods (e.g., IEF and HPLC or HPLC and CE). Whereas carriers of heterozygous β-thalassemia are not detectable in newborns by measurement of HbA_2_, the measurement of HbA and a cut-off <15% may be indicative for the β-thalassemia trait in newborns [19]. Confirmatory diagnostic follow-ups using a new sample with blood count, hemoglobin separation analysis, and, if necessary, DNA analysis then provide a definite result. These inexpensive methods can be used for carrier detection to improve family health, as well as in states where no antenatal screening program is in place; however, they can also lead to stigmatization and counterproductive effects [26,27,28].

## 4. Pre-Analytics

Limitations of the aforementioned methods lie often in the pre-analytical phase. The used material can be fresh cord blood or dried blood spot from a heel prick. Storage of cord blood should not exceed seven days at 4 °C and that of dried blood spots should not exceed 1 month. Older samples show lower elution of samples from the DBS and, often, the degradation of hemoglobin, resulting in a high baseline, increased noise, and unclear peaks with difficulties in zone adjustment and quantification, especially in Hb species present at low percentage.

Blood from premature babies contains lower percentages of HbA or other β-chain variants, which may lead to false negative results in screening for SCD. Although an exact gestational age for requesting a second sample is not defined, premature babies under 33 weeks of gestational age should receive repeated testing.

Babies who received blood transfusions show increased percentages of adult hemoglobin HbA and HbA_2_ and the presence of a severe hemoglobinopathy may be masked. Therefore, it seems essential that transfused babies can be identified using either HbA levels above the 2.5 multiples of median (MoM) dependent on gestational age or ratios of HbF/HbA [12].

## 5. Conclusions

HPLC, CE, and IEF have proven to be suitable methods for NBS for sickle cell disease and other hemoglobinopathies in several different states and regions with specific legal and economic conditions. They have very high sensitivity and high specificity regarding SCD with patterns of FS, FSC, and FSE, and just slightly lower for patterns of FSA and FSD. Other non-sickling hemoglobinopathies were successfully included in newborn screening programs, and future developments must match the achievements of this recent era of hemoglobin analysis systems and will add new flexibility.

## Figures and Tables

**Table 1:** Hemoglobinopathies relevant in newborn screening (NBS) and their respective patterns.

Target Disease	Pattern	
Sickle cell disease Primary target		
HbSS, HbS/β^0^ thalassemia, HbS/δβ thalassemia, HbS/HPFH	FS ^1^	
HbS/C	FSC	
HbS/β^+^ thalassemia	FSA	
HbS/D^Punjab^	FSD ^2^	
HbS/E	FSE	
HbS/other variant ^3^	FSX	
Thalassemia syndromes and other hemoglobinopathies		Secondary target
β^0^ thalassemia	F only	
β^+^ thalassemia	FA ^4^	
HbEE HbE/β^0^ thalassemia	FE	
HbE/β^+^ thalassemia	FEA	
HbCC or HbC/β^0^ thalassemia	FC	
HbC/β^+^ thalassemia	FCA	
HbDD^Punjab^ or HbD^Punjab^/β^0^ thalassemia	FD	
HbD^Punjab^/β^+^ thalassemia	FDA	
Hb variant not specified homozygous Hb variant/β^0^ or β^+^ thalassemia	FX FXA	
Severe α thalassemia, HbH disease	FABart’s ^5^	
Severe α thalassemia, HbH disease with other variants ^6^	FE Bart’s (e.g) FAX Bart’s	
Traits Secondary target		
HbS	FAS	
HbD^Punjab^	FAD	
HbC	FAC	
HbE	FAE	
Other Hb variant ^7^	FAX	
Normal	FA	

^1^ Hb variants migrating similarly to HbS may be hidden; ^2^ HbD has to be verified as HbD^Punjab^, as many other variants run like HbD^Punjab^; ^3^ Hb variants not specified during screening, or specified with second-line methods, e.g., Hb Lepore, or HbO^Arab^ if included in the program; ^4^ HbA <5% or <1.5% (overlap of premature newborns and normal newborns) depend on cut-off used; ^5^ detected Hb Bart’s (over cut-off or qualitative); ^6^ includes detected Hb Bart’s with combined α- or β-variants and, e.g., HbE with Hb Bart’s; ^7^ Hb variants not specified during screening or specified with second-line methods, e.g., Hb Lepore, or HbO^Arab^ if included in the program.

## References

[1] Serjeant B.E., Forbes M., Williams L.L., Serjeant G.R. (1974). Screening cord bloods for detection of sickle cell disease in Jamaica. Clin. Chem..

[2] Pearson H.A., O’Brien R.T., McLntosh S., Aspnes G.T., Yang M. (1974). Routine screening of umbilical cord blood for sickle cell diseases. JAMA.

[3] Goldenberg A.J., Comeau A.M., Grosse S.D., Tanksley S., Prosser L.A., Ojodu J., Botkin J.R., Kemper A.R., Green N.S. (2016). Evaluating Harms in the Assessment of Net Benefit: A Framework for Newborn Screening Condition Review. Matern. Child Health J..

[4] Eastman J.W., Wong R., Liao C.L., Morales D.R. (1996). Automated HPLC screening of newborns for sickle cell anemia and other hemoglobinopathies. Clin. Chem..

[5] Campbell M., Henthorn J.S., Davies S.C. (1999). Evaluation of cation-exchange HPLC compared with isoelectric focusing for neonatal hemoglobinopathy screening. Clin. Chem..

[6] Bouva M.J., Mohrmann K., Brinkman H.B., Kemper-Proper E.A., Elvers B., Loeber J.G., Verheul F.E., Giordano P.C. (2010). Implementing neonatal screening for haemoglobinopathies in the Netherlands. J. Med. Screen..

[7] Frommel C., Brose A., Klein J., Blankenstein O., Lobitz S. (2014). Newborn screening for sickle cell disease: Technical and legal aspects of a German pilot study with 38,220 participants. BioMed Res. Int..

[8] Greene D.N., Pyle A.L., Chang J.S., Hoke C., Lorey T. (2012). Comparison of Sebia Capillarys Flex capillary electrophoresis with the BioRad Variant II high pressure liquid chromatography in the evaluation of hemoglobinopathies. Clin. Chim. Acta.

[9] Lorey F., Cunningham G., Shafer F., Lubin B., Vichinsky E. (1994). Universal screening for hemoglobinopathies using high-performance liquid chromatography: Clinical results of 2.2 million screens. Eur. J. Hum. Genet..

[10] Hoppe C.C. (2011). Newborn screening for hemoglobin disorders. Hemoglobin.

[11] Upadhye D.S., Jain D.L., Trivedi Y.L., Nadkarni A.H., Ghosh K., Colah R.B. (2014). Newborn screening for haemoglobinopathies by high performance liquid chromatography (HPLC): Diagnostic utility of different approaches in resource-poor settings. Clin. Chem. Lab. Med..

[12] Allaf B., Patin F., Elion J., Couque N. (2018). New approach to accurate interpretation of sickle cell disease newborn screening by applying multiple of median cutoffs and ratios. Pediatr. Blood Cancer.

[13] Renom G., Mereau C., Maboudou P., Perini J.M. (2009). Potential of the Sebia Capillarys neonat fast automated system for neonatal screening of sickle cell disease. Clin. Chem. Lab. Med..

[14] Wild B. The Haemoglobinopathies: Learning from EQA, UK NEQAS. https://www.ukneqash.org/documents.php.

[15] Serjeant G.R., Serjeant B.E., Hambleton I.R., Oakley M., Thein S.L., Clark B. (2017). A Plea for the Newborn Diagnosis of Hb S-Hereditary Persistence of Fetal Hemoglobin. Hemoglobin.

[16] Hustace T., Fleisher J.M., Sanchez Varela A.M., Podda A., Alvarez O. (2011). Increased prevalence of false positive hemoglobinopathy newborn screening in premature infants. Pediatr. Blood Cancer.

[17] Moradkhani K., Riou J., Wajcman H. (2013). Pitfalls in the genetic diagnosis of Hb S. Clin. Biochem..

[18] Ryan K., Bain B.J., Worthington D., James J., Plews D., Mason A., Roper D., Rees D.C., de la Salle B., Streetly A. (2010). Significant haemoglobinopathies: Guidelines for screening and diagnosis. Br. J. Haematol..

[19] Mantikou E., Arkesteijn S.G., Beckhoven van J.M., Kerkhoffs J.L., Harteveld C.L., Giordano P.C. (2009). A brief review on newborn screening methods for hemoglobinopathies and preliminary results selecting beta thalassemia carriers at birth by quantitative estimation of the HbA fraction. Clin. Biochem..

[20] Bouva M.J., Sollaino C., Perseu L., Galanello R., Giordano P.C., Harteveld C.L., Cnossen M.H., Schielen P.C., Elvers L.H., Peters M. (2011). Relationship between neonatal screening results by HPLC and the number of alpha-thalassaemia gene mutations; consequences for the cut-off value. J. Med. Screen..

[21] Jindatanmanusan P., Riolueang S., Glomglao W., Sukontharangsri Y., Chamnanvanakij S., Torcharus K., Viprakasit V. (2014). Diagnostic applications of newborn screening for alpha-thalassaemias, haemoglobins E and H disorders using isoelectric focusing on dry blood spots. Ann. Clin. Biochem..

[22] Liao C., Zhou J.Y., Xie X.M., Tang H.S., Li R., Li D.Z. (2014). Newborn screening for Hb H disease by determination of Hb Bart’s using the Sebia capillary electrophoresis system in southern China. Hemoglobin.

[23] Uaprasert N., Settapiboon R., Amornsiriwat S., Sarnthammakul P., Thanapat T., Rojnuckarin P., Sutcharitchan P. (2014). Diagnostic utility of isoelectric focusing and high performance liquid chromatography in neonatal cord blood screening for thalassemia and non-sickling hemoglobinopathies. Clin. Chim. Acta.

[24] Rugless M.J., Fisher C.A., Stephens A.D., Amos R.J., Mohammed T., Old J.M. (2006). Hb Bart’s in cord blood: An accurate indicator of alpha-thalassemia. Hemoglobin.

[25] Wu M.Y., Xie X.M., Li J., Li D.Z. (2015). Neonatal screening for alpha-thalassemia by cord hemoglobin Barts: How effective is it?. Int. J. Lab. Hematol..

[26] Bain B.J. (2011). Haemoglobinopathy diagnosis: Algorithms, lessons and pitfalls. Blood Rev..

[27] Bombard Y., Miller F.A., Hayeems R.Z., Wilson B.J., Carroll J.C., Paynter M., Little J., Allanson J., Bytautas J.P., Chakraborty P. (2012). Health-care providers’ views on pursuing reproductive benefit through newborn screening: The case of sickle cell disorders. Eur. J. Hum. Genet..

[28] Ross L.F. (2012). Newborn screening for sickle cell disease: Whose reproductive benefit?. Eur. J. Hum. Genet..

